# Circular RNA circCCDC85A inhibits breast cancer progression via acting as a miR-550a-5p sponge to enhance MOB1A expression

**DOI:** 10.1186/s13058-021-01497-6

**Published:** 2022-01-04

**Authors:** Lingjiao Meng, Sheng Chang, Yang Sang, Pingan Ding, Liuxin Wang, Xixi Nan, Ruiyu Xu, Fei Liu, Lina Gu, Yang Zheng, Ziyi Li, Meixiang Sang

**Affiliations:** 1 Tumor Research Institute, The Fourth Affiliated Hospital of Hebei Medical University, Shijiazhuang, Hebei 050017 People’s Republic of China; 2 Research Center, The Fourth Hospital of Hebei Medical University, Shijiazhuang, Hebei 050017 People’s Republic of China; 3 Animal Center, The Fourth Hospital of Hebei Medical University, Shijiazhuang, Hebei 050017 People’s Republic of China; 4 The Third Department of Surgery, The Fourth Hospital of Hebei Medical University, Shijiazhuang, Hebei 050017 People’s Republic of China

**Keywords:** Breast cancer, Circular RNA, circCCDC85A, miR-550a-5p, MOB1A

## Abstract

**Background:**

A growing body of evidence indicates that abnormal expression of circular RNAs (circRNAs) plays a crucial role by acting as molecular sponges of microRNAs (miRNAs) in various diseases, including cancer. In this study, we explored whether circCCDC85A could function as a miR-550a-5p sponge and influence breast cancer progression.

**Methods:**

We detected the expression of circCCDC85A in breast cancer tissues and cells using fluorescence in situ hybridization (FISH) and quantitative reverse transcription polymerase chain reaction (qRT-PCR). CCK-8 and colony formation assay were used to detect the proliferative ability of breast cancer cells. Wound healing assay and transwell migration and invasion assays were used to detect the migrative and invasive abilities of breast cancer cells. We also examined the interactions between circCCDC85A and miR-550a-5p using FISH, RNA-binding protein immunoprecipitation (RIP), and luciferase reporter assay. Moreover, we performed luciferase reporter assay, qRT-PCR, and Western blot to confirm the direct targeting of miR-550a-5p to MOB1A.

**Results:**

The expression of circCCDC85A in breast cancer tissues was obviously lower than that in normal breast tissues. Over-expression of circCCDC85A substantially inhibited the proliferative, migrative, and invasive ability of breast cancer cells, while knocking down of circCCDC85A enhanced the aforementioned properties of breast cancer cells. Moreover, enforced expression of circCCDC85A inhibits the oncogenic activity of miR-550a-5p and increases the expression of MOB1A targeted by miR-550a-5p. Further molecular mechanism research showed that circCCDC85A may act as a molecular sponge for miR-550a-5p, thus restoring miR-550a-5p-mediated targeting repression of tumor suppressor MOB1A in breast cancer cells.

**Conclusion:**

Our findings provide novel evidence that circCCDC85A inhibits the progression of breast cancer by functioning as a molecular sponge of miR-550a-5p to enhance MOB1A expression.

**Supplementary Information:**

The online version contains supplementary material available at 10.1186/s13058-021-01497-6.

## Background

Breast cancer is one of the most prevalent malignant tumor in women, accounting for the fifth highest rate of mortality among women [1]. China has a high incidence of breast cancer, with an increasing incidence seen in younger women. The occurrence and progression of breast cancer are intricate pathological processes. Various oncoproteins and tumor suppressor proteins have been known to affect the development and progression of breast cancer [2–5]. Increasing studies now report that several types of molecules, such as non-coding RNAs (ncRNAs), also play a crucial role in these processes [6].

As a new special cluster of RNAs with a closed loop structure, circular RNAs (circRNAs) do not contain a 5′ end cap and a 3′ end poly (A) tail [7]. The unique closed-loop structure of circRNAs make them tolerant towards the digestion of exonuclease RNase R and ensure that they are stably expressed in the cell, to enable them to play a long-term role in the regulation of transcription [8]. In recent years, accumulating evidence, involving high-throughput RNA sequencing (RNA-Seq), has identified several types of circRNAs and gradually uncovered the mystery of its function in eukaryotic cells [9, 10]. So far, it has been found that circRNAs are involved in the progression of various tumors by serving as microRNA (miRNA) sponges, translating polypeptides or proteins, interacting with proteins, and regulating gene transcription and splicing processes [11–15]. Several studies have shown that some exon-derived circRNAs, such as ciRS-7, circ-Sry, circ-ITCH, and circHIPK3, are rich in miRNA response elements (MRE) and could act as miRNA sponges through competitive endogenous RNA (ceRNA) mechanisms to competitively bind miRNAs, thereby further resulting in the negative regulation of miRNAs and their downstream target genes [9, 11, 16–20].

Recently, emerging studies have demonstrated that circRNAs play an essential role in the progression of breast cancer [21–39]. In this study, we identified the role of circRNA circCCDC85A (hsa_circ_0120472) that was downregulated in breast cancer tissues and cells. We found that the overexpression of circCCDC85A inhibited the proliferative, migrative, and invasive ability of breast cancer cells. Moreover, we demonstrated that circCCDC85A could inhibit the oncogenic activity of miR-550a-5p and increase the expression of MOB1A targeted by miR-550a-5p. This study provided novel evidence that circCCDC85A inhibits the progression of breast cancer by functioning as a molecular sponge of miR-550a-5p to enhance MOB1A expression.

## Methods

### Clinical specimens

We selected 58 breast cancer tissues and 40 normal breast tissues from the Fourth Hospital of Hebei Medical University. No patient included in this study underwent any preoperative chemotherapy and radiotherapy. This study was approved by the Medical Ethics Committee of our hospital, and informed consent was obtained from all patients prior to the study.

### Animal experiment

For the xenograft tumor experiment, four-week-old female BALB/c nude mice were randomly divided into four groups (*n* = 4 for each group). We subcutaneously injected the mice with MDA-MB-231 cells (5 × 10^6^ cells per mouse) that were stably transfected with either (1) pLC5-ciR + miR-NC; (2) pLC5-ciR + miR-550a-5p; (3) circCCDC85A + miR-NC; or (4) circCCDC85A + miR-550a-5p. After tumor formation, we examined the volume of the tumor every three days to evaluate tumor growth. After three weeks, we sacrificed the mice and analyzed the tumor weight. The animal experiments were approved by the Animal Care Committee of our hospital.

### Vector construction

To construct circCCDC85A over-expression vector, we amplified hsa_circ_0120472 sequence and then cloned it into the pLC5-ciR empty vector (Geneseed Biotech Corporation, China). For the circCCDC85A reporter vector, the linear sequence of circCCDC85A was amplified and then cloned into psiCHECK2 empty vector (Geneseed Biotech Corporation, China). For MOB1A 3’UTR vector, we synthesized the 3’UTR wild type or mutant sequence of MOB1A and then cloned it into the pGL3 empty vector (Promega, USA).

### Fluorescence in situ hybridization (FISH)

The biotin-labeled circCCDC85A probe and digoxin-labeled miR-550a-5p probe were designed and synthesized by the QIAGEN company (Germany). We cultured MDA-MB-231 and MCF-7 cells on coverslips overnight, and fixed them with 4% paraformaldehyde. After prehybridization in phosphate-buffered saline with 0.5% Triton X-100, cells were hybridized with circCCDC85A and miR-550a-5p probes overnight at 37 °C. The following day, circCCDC85A fluorescence signal was detected by Cy5-streptavidin conjugate kit (Invitrogen), and miR-550a-5p fluorescence signal was detected by anti-digoxin antibody (Abcam) and Tyramide SuperBoost kit with Alexa Fluor 488 (Invitrogen). Tissue slides were deparaffinized in xylene and hydrated in ethanol. The subsequent pre-hybridization and circCCDC85A hybridization procedures were the same as above. The nuclei were stained by DAPI for 10 min and the signal images were observed by LSM 900 confocal microscope (ZEISS, Germany).

### Cell culture

We incubated human breast cancer cell lines (MDA-MB-231, BT-549, MCF-7) in DMEM media (GIBCO, USA) with 10% heat-inactivated (56 °C) fetal bovine serum (GIBCO, USA) and 1% penicillin–streptomycin solution (100X). We cultured the cells in a sterile incubator containing 5% CO_2_ in air at 37 °C.

### DNA and RNA extraction and RNase R treatment

We extracted genomic DNA from breast cancer tissues and cells using a DNA extraction kit (TIANGEN, China), according to the manufacturer’s instructions. We extracted total RNA from breast cancer tissues and cells with TRIzol Reagent (Invitrogen, USA) based on the standard protocol. For the RNase R treatment assay, we treated the total RNA with RNase R from *E. coli* (Epicentre, USA) for 15 min at 37 °C and 3 min at 85 °C using a concentration of 3 U/μg RNA; next, we checked the efficacy of RNase R treatment using quantitative reverse transcription polymerase chain reaction (qRT-PCR). We extracted RNA from each well, reverse transcribed it into cDNA, and then detected the expression of circCCDC85A and linear CCDC85A using qRT-PCR.

### qRT-PCR and the nucleic acid agarose gel electrophoresis

We used the GoScript Reverse Transcription System kit (Promega, USA) for preparing cDNA from total RNA based on the standard manual. The PCR reaction was carried out by using GoTaq qPCR Master Mix (Promega, USA) in Applied Biosystems fluorescence quantitative PCR instrument. The thermal protocol was set to pre-denaturation at 95 °C for 5 min, denaturation at 95 °C for 15 s, annealing at 58 °C for 30 s, extension at 72 °C for 30 s, a total of 40 cycles, and extension at 72 °C for 10 min. The primers used in the PCR amplification are listed in Additional file 3: Table S1. Bulge-loop miRNA RT primer and qRT-PCR primer specific for miR-550a-5p were synthesized by RiboBio Corporation (Guangzhou, China). We used the 2^−ΔΔCT^ method to calculate the relative expression level. The ordinary RT-PCR products were separated using 2% agarose gel and the gray scale of the band was then detected using UV irradiation.

### CCK-8 assay

We seeded breast cancer cells of the experimental and control groups in a 96-well plate at a density of 4 × 10^3^ cells per well. After cell attachment, we carried out the CCK-8 assay at five indicated time points. Briefly, we added 10 μl of CCK-8 reagent into each well. After a short vibration, we placed the cells in the incubator at 37 °C for 2 h. Next, we detected the absorbance value of the cell at 450 nm using microplate reader (Tecan, USA).

### Colony formation assay

We seeded breast cancer cells of the experimental and control groups in a 6-well plate at a density of 2 × 10^3^ cells per well. We then placed the cells in the incubator at 37 °C for approximately 10 days until dot clones were visible to the naked eye. Next, we fixed the cell clones with 4% paraformaldehyde and stained them with crystal violet. Finally, we photographed the cell clones of each well with a camera and counted them under a microscope.

### Wound healing assay

We seeded breast cancer cells of the experimental and control groups in a 6-well plate and scraped them with the end of 10 μl pipette tip to define as the 0 h time point. Subsequently, we photographed cell migration under at least five random microscope fields at the 0 h and 24 h time points. The migration distance across the diminished injury of the cells in each group was measured and normalized to the 0 h time point.

### Transwell migration and invasion assay

We performed transwell migration and invasion assays using the transwell empty chamber (for migration detection) or pre-coated matrigel chamber (for invasion detection), based on the operation manual (BD Science, USA). We seeded breast cancer cells of the experimental and control groups at a density of 2 × 10^5^ cells per well in the upper transwell chambers in the 24-well plate and incubated them at 37 °C for 24–48 h. Next, we fixed the cells in the chamber with 4% paraformaldehyde and stained them with crystal violet. Finally, we counted the migration and invasion cell numbers under at least five random microscopic fields.

### RNA-binding protein immunoprecipitation (RIP)

Myc-tagged AGO2 vector and miR-550a-5p mimics were co-transfected into the breast cancer cells. We carried out RIP assay using the Magna RIP™ RNA-Binding Protein Immunoprecipitation Kit (Millipore, USA) 48 h after co-transfection, based on the operation manual. Briefly, we lysed breast cancer cells by RIP Lysis Buffer in the kit, supplemented with RNase inhibitor and proteinase inhibitor cocktail. We added the cell lysate with anti-Myc (Abcam, ab9106) or anti-IgG control coupled Magnetic Beads Protein A/G and then rotated it overnight at 4 °C. The next day, we washed the beads six times with cold RIP wash buffer and extracted the RNA using TRIzol reagent. After purification, we performed qRT-PCR on the RNA sample using specific RIP primers to detect the presence of relative binding targets.

### Luciferase reporter assay

We seeded breast cancer cells in a 12-well plate at a density of 5 × 10^4^ cells per well and incubated them overnight until the cells were attached. We prepared the cell lysates 48 h after co-transfection using the luciferase reporter gene assay kit (Promega, USA), and we measured the luciferase activities of each group using Tecan Spark Multi-functional Microplate Reader (TECAN, Switzerland), based on the manual of dual luciferase reporter gene assay system (Promega, USA).

### Western blot

We performed a Western blot assay, according to the standard protocol. We collected breast cancer cells and centrifuged them at a low temperature. We then extracted the cell total protein using RIPA lysis buffer coupled with 1% PMSF to inhibit protease activity. The obtained protein was denatured using high temperature and then separated by 10% SDS-PAGE electrophoresis. We then transferred the protein on the gel to the PVDF membrane (Millipore, USA). We applied rabbit polyclonal MOB1A antibody (Proteintech, Catalog Number: 12790-1-AP) and β-actin antibody (Proteintech, Catalog Number: 20536-1-AP) at 1:1000 dilution for the detection of immunoreactivity. Lastly, the PVDF membranes were imaged by using the ECL Plus solution (Solarbio, China), based on the manual’s instructions.

### Bioinformatics analysis

We predicted the potential circCCDC85A binding miRNA using the CircNet database (http://circnet.mbc.nctu.edu.tw/) [40]. We carried out target gene prediction of miR-550a-5p using the DIANA TOOLS—miRPath v3.0 (http://www.microrna.gr/miRPathv3) [41]. We used the Kyoto Encyclopedia of Genes and Genomes (KEGG) pathway analysis for identifying differentially expressed mRNA enriched pathways of miR-550a-5p in TarBase v7.0 database by DIANA TOOLS. We performed all searches in accordance with the instructions of the online databases.

### Statistical analysis

We used the SPSS 22.0 software (SPSS Inc, USA) to evaluate the statistical significance of all experiments in this study. Quantitative data were analyzed using Student's t-test or ANOVA, and qualitative data were analyzed using the Chi-squared test. All statistical analyses adopted two-tailed test, and a *P* value of < 0.05 was considered significant.

## Results

### Circular RNA circCCDC85A is downregulated in breast cancer

**Fig. 1:**
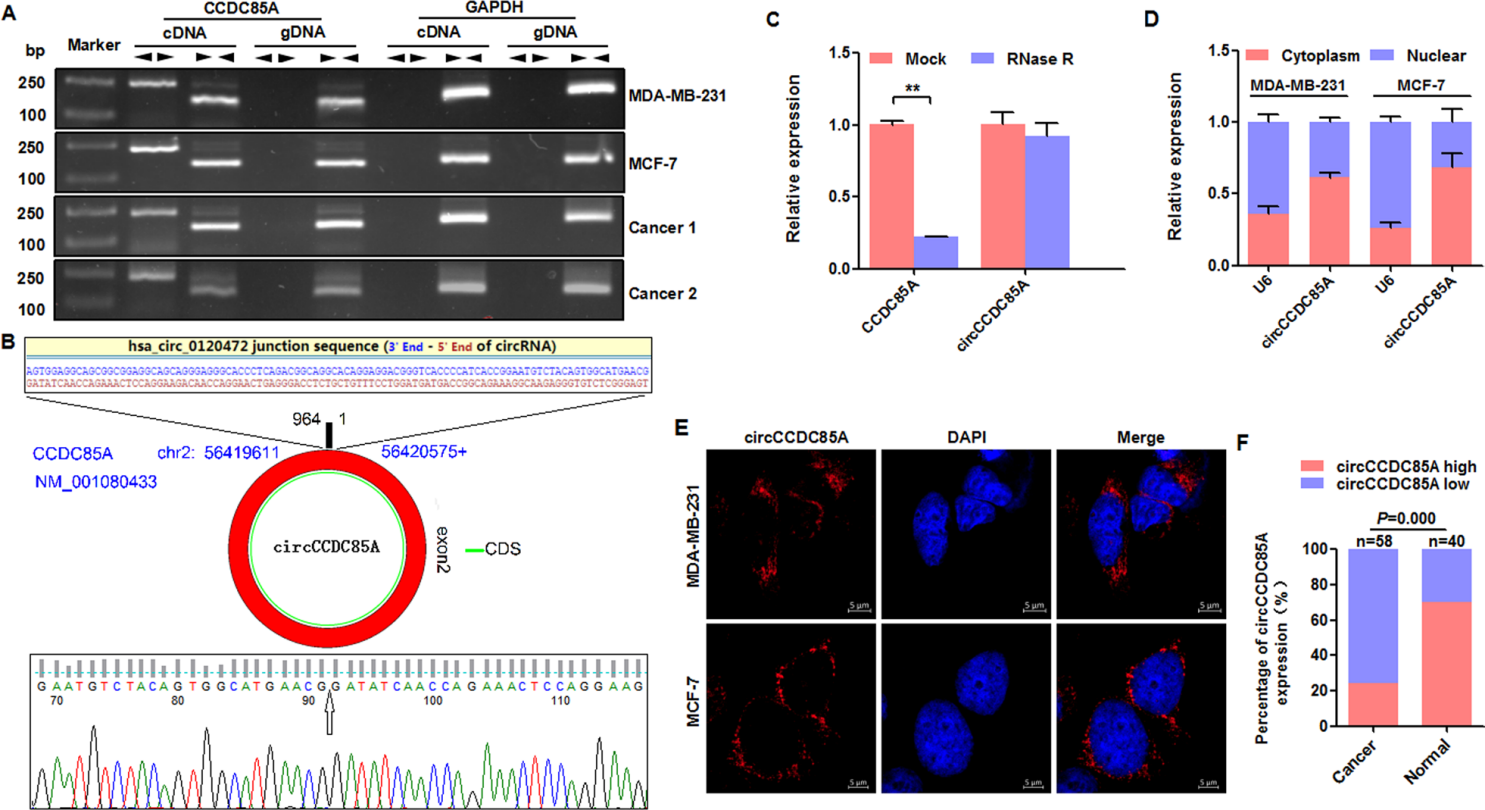
Circular RNA circCCDC85A is downregulated in breast cancer. **A** The existence of circCCDC85A was validated in two breast cancer cells and two cases of breast cancer tissues by RT-PCR. Divergent primers amplified circular RNA transcript circCCDC85A in cDNA but not gDNA. While convergent primers amplified linear RNA transcript CCDC85A in both cDNA and gDNA. GAPDH was used as an internal reference. **B** Sanger sequencing result of the above RT-PCR product indicated the junction sequence of circCCDC85A. **C** The expression level of circCCDC85A and the linear CCDC85A were detected by qRT-PCR in MCF-7 cells with RNase R treatment. **D** The relative expression level of circCCDC85A in cytoplasm and nuclear by qRT-PCR after fractionation of nuclear and cytoplasmic RNA in MDA-MB-231 and MCF-7 cells. **E** FISH assay showed that circCCDC85A preferentially localized in the cytoplasm of MDA-MB-231 and MCF-7 cells. **F** The percentage of circCCDC85A expression in 58 breast cancer tissues and 40 normal breast tissues. **P < 0.01

Circular RNA circCCDC85A, annotated as hsa_circ_0120472 in the circBase database, is transcribed from the exon 2 of the host gene *CCDC85A,* which is located on chromosome 2. In this study, we designed and synthesized two sets of PCR primers for CCDC85A, of which the divergent primers were used to amplify the circular transcript and the convergent primers were used to amplify the related linear transcript. The cDNA obtained using reverse transcription reaction and gDNA extracted from the genome in two breast cancer cell lines (MDA-MB-231 and MCF-7) and two cases of breast cancer tissues (Cancer 1 and Cancer 2) were applied as templates for PCR reaction. GAPDH was used as an internal reference gene. The results showed that the circular transcript of CCDC85A was amplified in the cDNA sample alone through divergent primers, but the linear transcript of CCDC85A was amplified in both cDNA and gDNA samples through convergent primers (Fig. 1A). Then, the junction sequence of the circular transcript circCCDC85A was verified using Sanger sequencing (Fig. 1B). To further detect the stability of circCCDC85A, the total RNA of MCF-7 cells was carried out for RNase R treatment, and the qRT-PCR results indicated that circCCDC85A was resistant to RNase R digestion, but the related linear CCDC85A was decreased after treatment with RNase R (Fig. 1C). Moreover, the relative expression level of circCCDC85A in the cytoplasm was higher than that in the nucleus by qRT-PCR after fractionation of nuclear and cytoplasmic RNA in MDA-MB-231 and MCF-7 cells (Fig. 1D). Consistently, FISH assay showed that circCCDC85A preferentially localized in the cytoplasm of MDA-MB-231 and MCF-7 cells (Fig. 1E). Next, we detected the expression of circCCDC85A in 58 breast cancer tissues and 40 normal breast tissues; the FISH analysis revealed that the expression of circCCDC85A was significantly lower in breast cancer tissues than that in normal breast tissues (Fig. 1F). The above results indicated that circCCDC85A is downregulated in breast cancer.

### circCCDC85A inhibits the proliferation, migration, and invasion of breast cancer cells

**Fig. 2:**
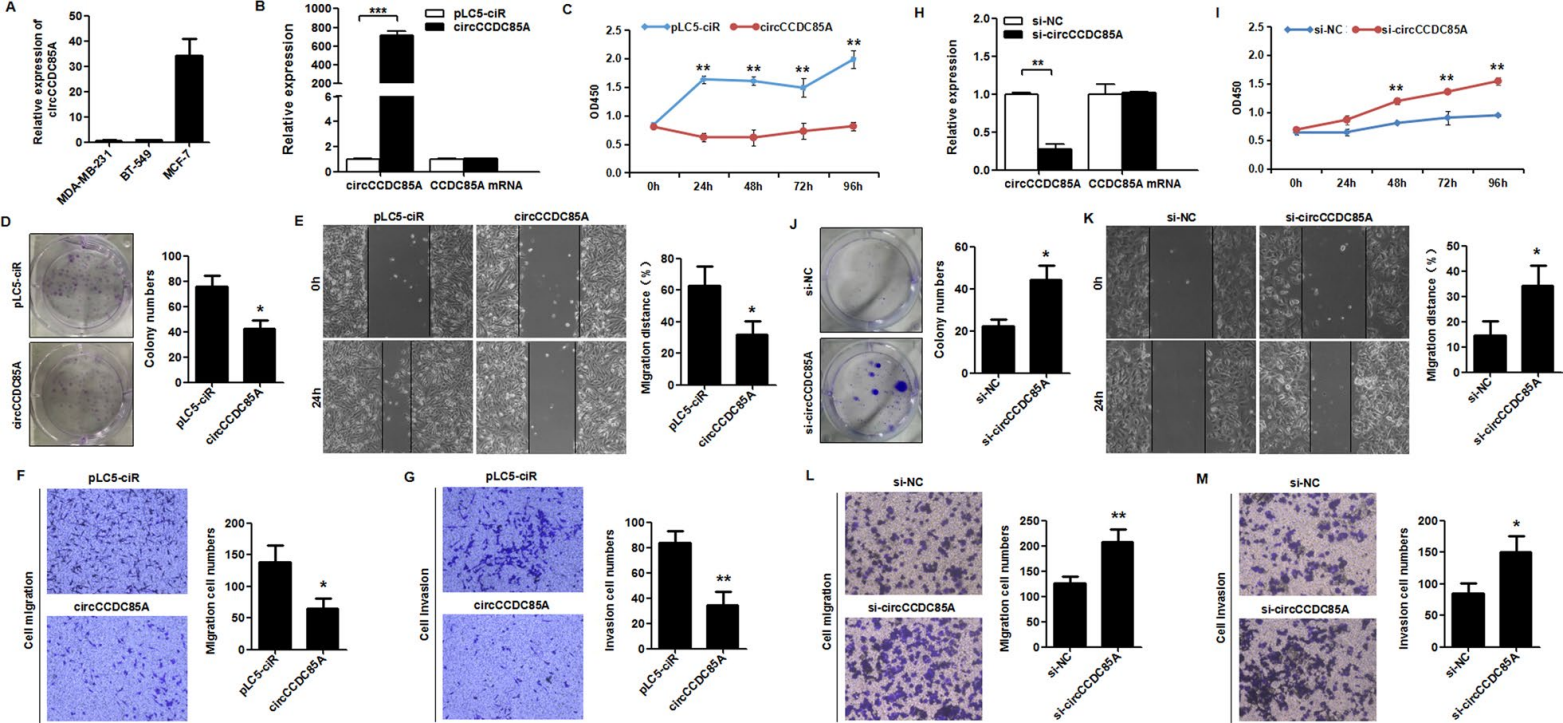
circCCDC85A inhibits proliferation, migration and invasion of breast cancer cells. **A** The expression of circCCDC85A in three breast cancer cell lines was detected by qRT-PCR. **B** The expression of circCCDC85A and linear CCDC85A mRNA in MDA-MB-231 cells after transfection with circCCDC85A or pLC5-ciR empty vector were detected by qRT-PCR. **C**, **D** The proliferation viability and colony formation ability of MDA-MB-231 cells transfected with circCCDC85A or pLC5-ciR empty vector were evaluated by CCK-8 assay and colony formation assay. **E**–**G** The migration and invasion abilities of MDA-MB-231 cells transfected with circCCDC85A or pLC5-ciR empty vector were evaluated by wound healing assay, transwell migration and invasion assay. **H** The knocking down efficiency of circCCDC85A siRNA in MCF-7 cells was examined by qRT-PCR. **I**, **J** The proliferation viability and colony formation ability of MCF-7 cells transfected with circCCDC85A siRNA or NC siRNA were evaluated by CCK-8 assay and colony formation assay. **K**–**M** The migration and invasion abilities of MCF-7 cells transfected with circCCDC85A siRNA or NC siRNA were evaluated by wound healing assay, transwell migration and invasion assay. *P < 0.05, **P < 0.01, ***P < 0.001

To investigate the biological function of circCCDC85A in breast cancer cells, we first detected the expression level of circCCDC85A in three breast cancer cell lines using qRT-PCR. We found that circCCDC85A had the lowest expression level in MDA-MB-231 cells and the highest expression level in MCF-7 cells (Fig. 2A). Then, MDA-MB-231 cells were transfected with circCCDC85A over-expressing plasmid. The transfection efficiency of circCCDC85A in MDA-MB-231 cells was detected using qRT-PCR. It was shown that the level of circCCDC85A expression was increased after being transfected with circCCDC85A plasmid, but the expression level of CCDC85A mRNA did not significantly change (Fig. 2B). CCK-8 assay showed that the proliferative ability of MDA-MB-231 cells was significantly reduced after circCCDC85A transfection (Fig. 2C). Moreover, colony formation assay showed that the colony formation of circCCDC85A transfected MDA-MB-231 cells was inhibited as compared to the control group (Fig. 2D). Wound healing assay indicated that over-expression of circCCDC85A inhibited the migration ability of MDA-MB-231 cells after being transfected with circCCDC85A (Fig. 2E). Besides, transwell migration and invasion assay showed that over-expression of circCCDC85A suppressed the migration and invasive abilities of MDA-MB-231 cells (Fig. 2F, G). On the other hand, the circCCDC85A siRNA specifically targeting the junction site of circCCDC85A was transfected into MCF-7 cells. The knockdown efficiency of circCCDC85A in MCF-7 cells was tested by qRT-PCR. The results showed that the expression level of circCCDC85A was decreased after being transfected with si-circCCDC85A than with si-NC, but the expression level of CCDC85A mRNA did not change a lot (Fig. 2H). CCK-8 and colony formation assay showed that the proliferative ability of MCF-7 cells was obviously promoted after si-circCCDC85A transfection (Fig. 2I, J). Moreover, the wound healing and transwell assays showed that si-circCCDC85A enhanced the migration and invasive ability of MCF-7 cells after being transfected with si-circCCDC85A (Fig. 2K, L, M). The above findings revealed that circCCDC85A could inhibit the proliferative, migrative, and invasive abilities of breast cancer cells.

### circCCDC85A acts as a miR-550a-5p sponge in breast cancer cells

**Fig. 3:**
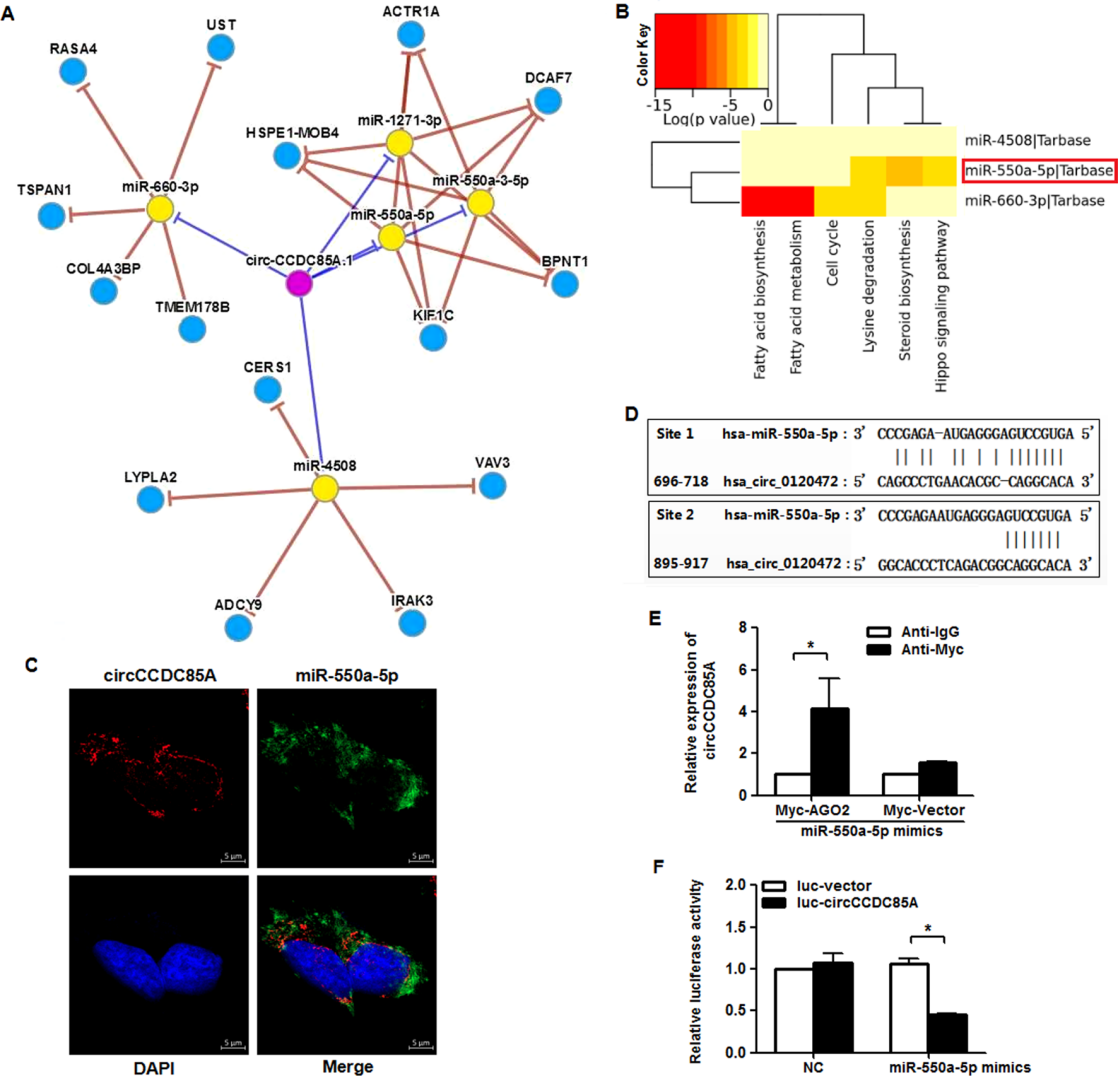
circCCDC85A acts as a miR-550a-5p sponge in breast cancer cells. **A** The potential miRNA targets of circCCDC85A were predicted by CircNet online database, and it was found that circCCDC85A may function as the sponge of miR-660-3p, miR-550a-5p and miR-4508. **B** The potential downstream pathways of the above three miRNAs were analyzed through tarbase database, and miR-550a-5p was found to be associated with the Hippo signaling pathway which is a well-known cancer-related pathway **C** FISH results showed that both circCCDC85A and miR-550a-5p were predominantly located in cytoplasm. **D** The miranda database was applied for prediction of the binding sites between circCCDC85A and miR-550a-5p, and it was found that there are two potential binding sites between hsa-miR-550a-5p and hsa_circ_0120472. **E** Immunoprecipitation of Myc-tagged AGO2 from MCF-7 cells transfected with either Myc-AGO2 or Myc-vector negative control plus miR-550a-5p mimics. The enrichment of circCCDC85A was detected by qRT-PCR. **F** Luciferase reporter assay was performed to detect the luciferase activity of luc-circCCDC85A or luc-vector negative control in MCF-7 cells co-transfected with miR-550a-5p mimics. *P < 0.05

The above evidence demonstrated that circCCDC85A may be abundantly expressed in breast cancer cells and is mainly localized in the cytoplasm, making it possible for circCCDC85A to act as a miRNA sponge. The bioinformatics prediction analysis of CircNet online database revealed that circCCDC85A may function as the sponge of miR-660-3p, miR-550a-5p, and miR-4508 (Fig. 3A). Next, we predicted the downstream pathways of these three miRNAs through the TarBase database. The results showed that miR-550a-5p may be associated with the Hippo signaling pathway which is a well-known cancer-related pathway (Fig. 3B). Moreover, a study has reported that the Hippo signaling pathway regulates cancer progression through circRNAs [42]. Therefore, we will focus on miR-550a-5p in the follow-up research. The FISH assay showed that both circCCDC85A and miR-550a-5p were predominantly located in the cytoplasm (Fig. 3C), which proved that circCCDC85A can potentially act as the molecular sponge of miR-550a-5p. The miRanda database was used for predicting the binding sites between circCCDC85A and miR-550a-5p, and we found that there are two potential binding sites between hsa-miR-550a-5p and hsa_circ_0120472 (Fig. 3D). Next, RIP assay was performed to verify the interactions between circCCDC85A and miR-550a-5p, and we found that circCCDC85A was significantly enriched by anti-Myc in circCCDC85A high-expressed MCF-7 cells co-transfected with Myc-AGO2 and miR-550a-5p mimics (Fig. 3E). To further detect the effect of miR-550a-5p on the activity of circCCDC85A, the luciferase reporter assay was performed in circCCDC85A high-expressed MCF-7 cells co-transfected with luc-circCCDC85A and miR-550a-5p mimics, and the results showed that miR-550a-5p significantly inhibited the luciferase activity of luc-circCCDC85A (Fig. 3F). The above data demonstrated that circCCDC85A may act as a miR-550a-5p sponge in breast cancer cells.

### miR-550a-5p promotes the proliferation, migration, and invasion of breast cancer cells

**Fig. 4:**
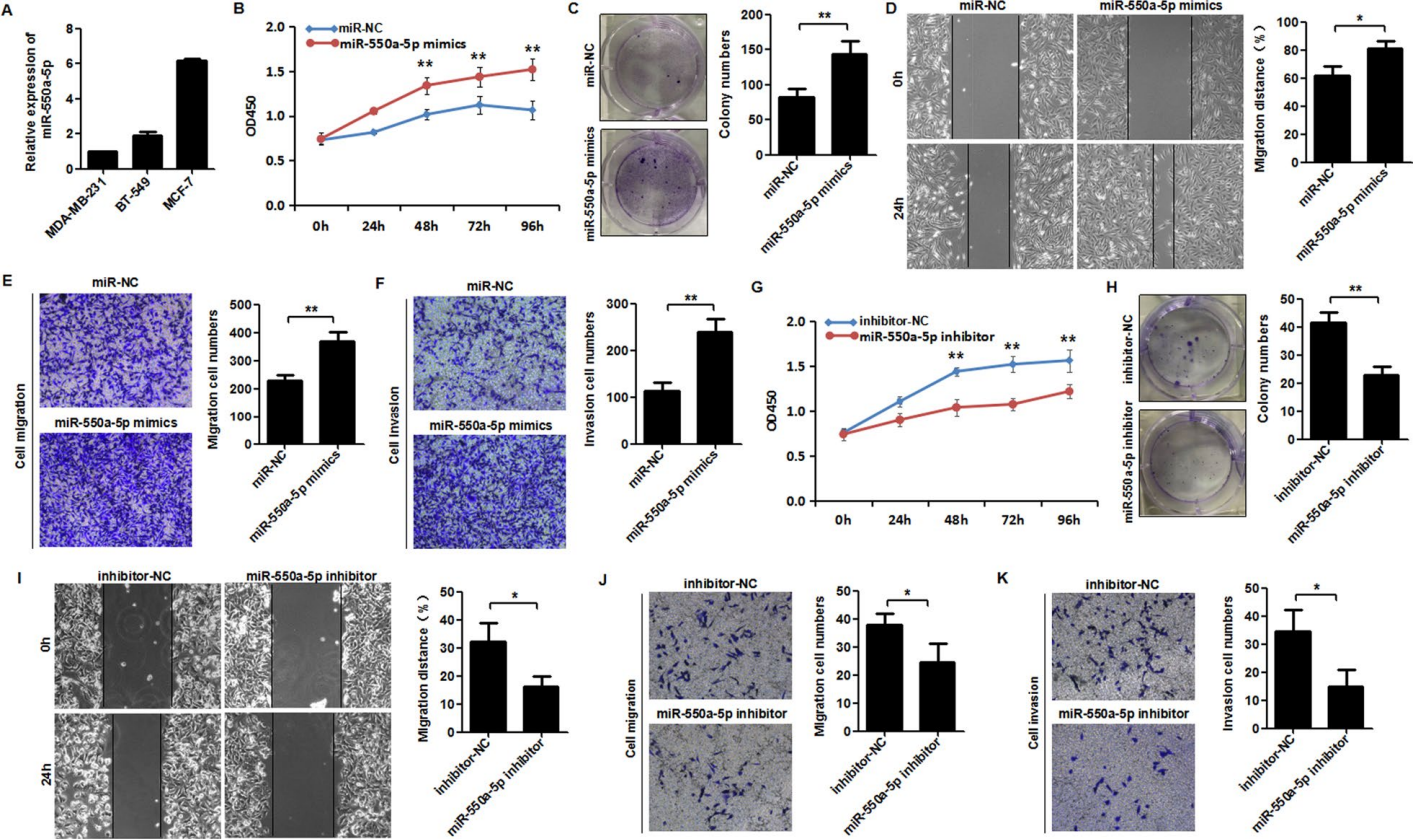
miR-550a-5p promotes proliferation, migration and invasion of breast cancer cells. **A** miR-550a-5p expression in three breast cancer cell lines was detected by qRT-PCR. **B, C**, The proliferation viability and colony formation ability of MDA-MB-231 cells transfected with miR-550a-5p mimics or miR-NC were evaluated by CCK-8 assay and colony formation assay. **D**–**F** The migration and invasion abilities of MDA-MB-231 cells transfected with miR-550a-5p mimics or miR-NC were evaluated by wound healing assay, transwell migration and invasion assay. **G, H** The proliferation viability and colony formation ability of MCF-7 cells transfected with miR-550a-5p inhibitor or inhibitor-NC were evaluated by CCK-8 assay and colony formation assay. **I**–**K** The migration and invasion abilities of MCF-7 cells transfected with miR-550a-5p inhibitor or inhibitor-NC were evaluated by wound healing assay, transwell migration and invasion assay. *P < 0.05, **P < 0.01

To verify the biological functions of miR-550a-5p in breast cancer cells, the expression of miR-550a-5p in three breast cancer cell lines was detected using qRT-PCR. It was shown that miR-550a-5p had the lowest expression level in MDA-MB-231 cells, and the highest expression level in MCF-7 cells (Fig. 4A). Then, MDA-MB-231 cells were transfected with miR-550a-5p mimics to detect the effects of miR-550a-5p overexpression on biological function. CCK-8 and colony formation assay showed that the proliferative ability of MDA-MB-231 cells was significantly reinforced after miR-550a-5p mimics transfection (Fig. 4B, C). Wound healing assay indicated that over-expression of miR-550a-5p enhanced the migration ability of MDA-MB-231 cells (Fig. 4D). Besides, the transwell migration and invasion assay also showed that the over-expression of miR-550a-5p enhanced the migrative and invasive abilities of MDA-MB-231 cells (Fig. 4E, F). On the other hand, the miR-550a-5p inhibitor was transfected into MCF-7 cells to inhibit the function of miR-550a-5p. CCK-8 and colony formation assay showed that the proliferative ability of MCF-7 cells was significantly decreased after miR-550a-5p inhibitor transfection (Fig. 4G, H). The wound healing assay and transwell assay indicated that miR-550a-5p inhibitor suppressed the migrative and invasive ability of MCF-7 cells (Fig. 4I, J, K). The above results suggested that miR-550a-5p could promote the proliferative, migrative, and invasive abilities of breast cancer cells.

### circCCDC85A exerts the repressive effect of proliferation, migration, and invasion in breast cancer cells via miR-550a-5p

**Fig. 5:**
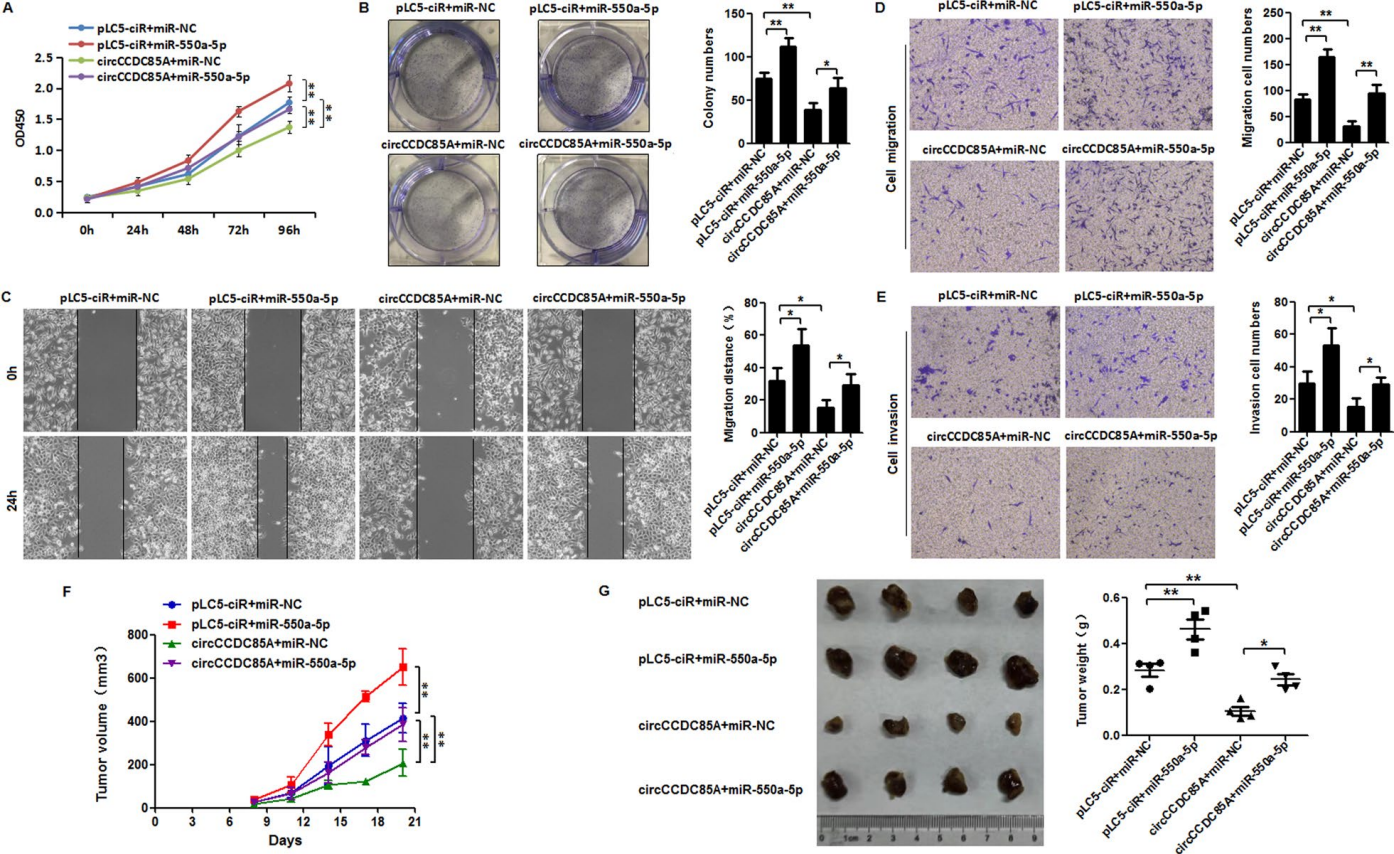
circCCDC85A exerts the repressive effect of proliferation, migration and invasion in breast cancer via miR-550a-5p. **A** and **B** CCK-8 and colony formation assay showed that miR-550a-5p over-expression alone increased the proliferation viability of MDA-MB-231 cells, but such enhanced effect was reversed when circCCDC85A added simultaneously. **C**–**E**, Wound healing assay, transwell migration and invasion assay showed that miR-550a-5p over-expression alone increased the migration and invasion viability of MDA-MB-231 cells, but the supplement of circCCDC85A offset such promotion. **F** and **G** Tumor growth and tumor weight were evaluated. *P < 0.05, **P < 0.01

To explore how circCCDC85A exerts the repressive biological function in breast cancer cells, MDA-MB-231 cells were transfected with miR-550a-5p, circCCDC85A, or the combination of miR-550a-5p and circCCDC85A; then, cell proliferative, migrative, and invasive capacities were detected. CCK-8 and colony formation assay indicated that miR-550a-5p over-expression alone increased the proliferation viability of MDA-MB-231 cells, but such an enhanced effect was reversed when circCCDC85A was added simultaneously (Fig. 5A, B). Similarly, over-expression of miR-550a-5p alone increased the migration and invasive viability of MDA-MB-231 cells, but the supplement of circCCDC85A offset such promotion (Fig. 5C, D, E). In vivo experiments in nude mice showed that the growth of xenograft tumor was promoted by enforced miR-550a-5p, but the enhancement was restored when circCCDC85A was supplemented (Fig. 5F, G). The above data revealed that circCCDC85A could exert the repressive effect of proliferation, migration, and invasion in breast cancer cells at least partially via miR-550a-5p.

### circCCDC85A restores miR-550a-5p mediated targeting repression of tumor suppressor MOB1A in breast cancer cells

**Fig. 6:**
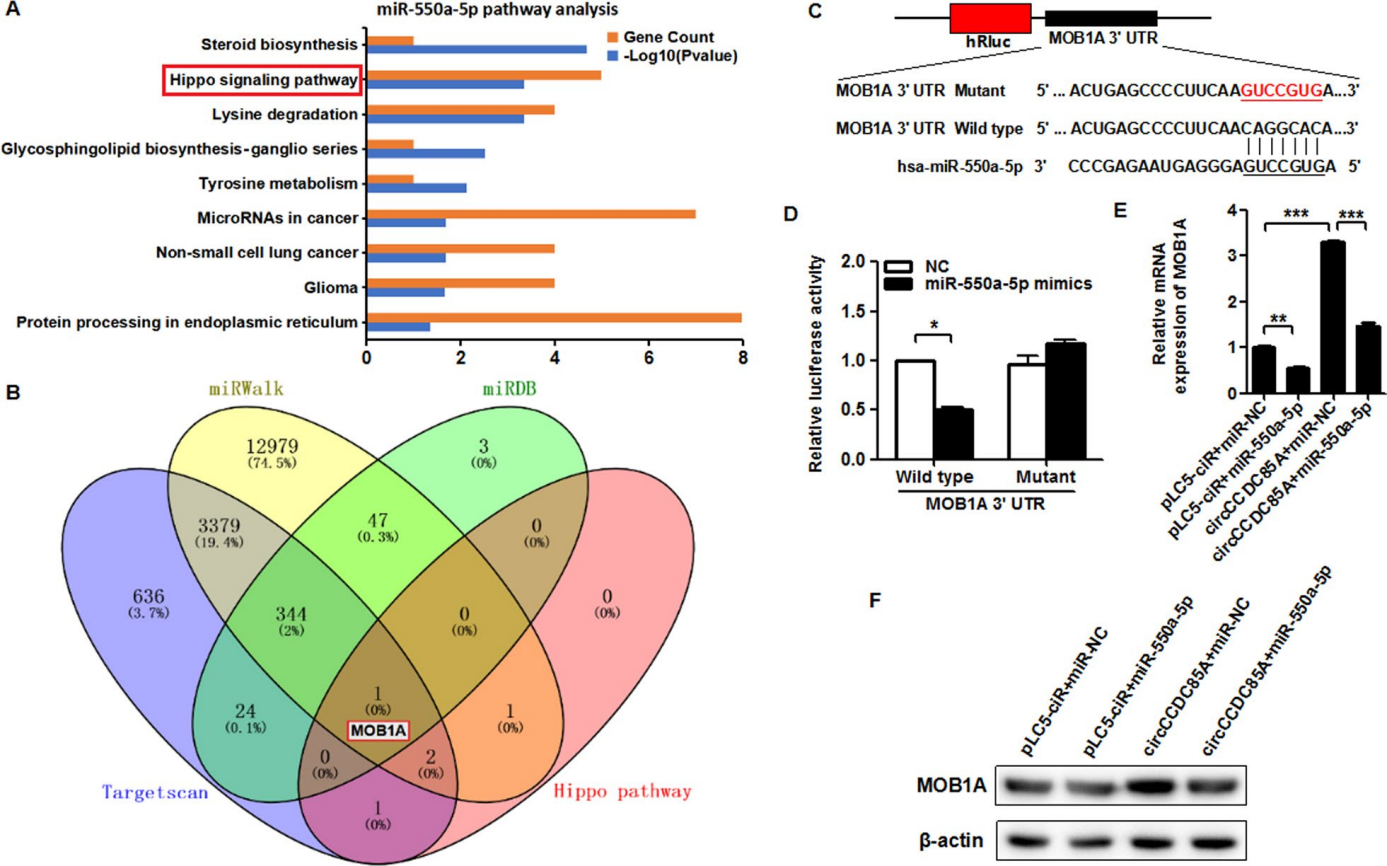
circCCDC85A restores miR-550a-5p mediated targeting repression of tumor suppressor MOB1A in breast cancer cells. **A** DIANA TOOLS was performed to predict the downstream signaling pathway of miR-550a-5p. **B** Targetscan, miRWalk and miRDB database were applied for prediction of the potential target genes of miR-550a-5p, and Venn diagram presented the intersection element of “Targetscan”, “miRWalk”, "miRDB" and "Hippo pathway". **C** The wild type 3’UTR and mutant 3’UTR of MOB1A were constructed into the human Renilla luciferase reporter vector. **D** Luciferase reporter assay was performed to detect the luciferase activity of wild type MOB1A 3’UTR vector or mutant MOB1A 3’UTR vector in MDA-MB-231 cells co-transfected with miR-550a-5p mimics or negative control vector. **E** MOB1A mRNA expression in MDA-MB-231 cells transfected with miR-550a-5p, circCCDC85A or miR-550a-5p plus circCCDC85A was detected by qRT-PCR. **F** MOB1A protein expression in MDA-MB-231 cells transfected with miR-550a-5p, circCCDC85A or miR-550a-5p plus circCCDC85A was detected by western blot. *P < 0.05, **P < 0.01, ***P < 0.001

To dissect the molecular mechanism of miR-550a-5p in breast cancer, DIANA TOOLS was performed to predict the downstream signaling pathway of miR-550a-5p. The results of the KEGG pathway analysis showed that the cancer-related Hippo signaling pathway was significantly enriched with miR-550a-5p in the TarBase database by DIANA TOOLS (Fig. 6A). The predicted KEGG pathway ID, name, P value, enriched gene count and gene name are shown in Additional file 1: Fig. S1. We next predicted the potential target genes of miR-550a-5p through Targetscan, miRWalk, and miRDB databases. The intersection element MOB1A of the common target genes of these three databases and the Hippo signaling pathway enriched genes was then screened for follow-up research (Fig. 6B). MOB1A reportedly functions as a tumor suppressor in several cancer types [43–46]. In this study, we enforced the expression of MOB1A in MDA-MB-231 cells using MOB1A overexpression plasmid (Additional file 2: Fig. S2A), and then detected cell proliferative, migrative, and invasive abilities. CCK-8 assay and colony formation assay revealed that MOB1A inhibited cell proliferation (Additional file 2: Fig. S2B, C). Wound healing assay, transwell migration, and invasion assay indicated that MOB1A inhibited cell migrative and invasive ability in breast cancer (Additional file 2: Fig. S2D, E, F).

To verify MOB1A as a direct target gene of miR-550a-5p, the wild type 3’UTR and mutant 3’UTR of MOB1A were constructed into the human Renilla luciferase reporter vector (Fig. 6C). Then the wild type or mutant 3’UTR of MOB1A was co-transfected with NC or miR-550a-5p mimics in MDA-MB-231 cells, and the luciferase reporter assay was performed. The results indicated that the luciferase activity of wild type MOB1A 3’UTR vector was significantly reduced, whereas it was hardly changed in the mutant MOB1A 3’UTR vector (Fig. 6D). The above evidence revealed that MOB1A was a direct target of miR-550a-5p. Then, we detected MOB1A mRNA and protein expression after transfecting miR-550a-5p mimics with or without circCCDC85A over-expression plasmid in MDA-MB-231 cells by qRT-PCR and western blot assay. The results showed that circCCDC85A rescued miR-550a-5p-mediated repression of MOB1A expression in MDA-MB-231 cells at both mRNA and protein levels (Fig. 6E, F). These data demonstrated that circCCDC85A as a molecular sponge of miR-550a-5p could restore miR-550a-5p-mediated MOB1A repression in breast cancer cells.

## Discussion

CircRNAs are abundant and stably expressed in eukaryotic cells, which potentially make them ideal biomarkers for various diseases. More importantly, there are several available circRNAs detected in human body fluids, such as blood, saliva, and exosomes, which may provide more options for clinical diagnosis and treatment. In breast cancer, accumulating circRNAs are linked with cell proliferation, apoptosis, metastasis, and chemotherapy sensitivity [47]. Therefore, exploring the role and mechanism of circRNAs in tumor progression will open up new avenues for future cancer treatment.

In this study, we identified the stability of circCCDC85A and its prevalent downregulation in breast cancer tissues. Enforced expression of circCCDC85A substantially inhibited the proliferative, migrative, and invasive ability of breast cancer cells, while knocking down of circCCDC85A enhanced those properties of breast cancer cells. Based on the above biological phenomenon, we further explored the molecular mechanism of circCCDC85A in breast cancer cells.

Thus far, circRNAs have been identified to influence biological functions in multiple ways, such as by serving as miRNA sponges, interacting with RNA binding proteins (RBPs), regulating transcription, translating peptides or proteins, and competing with pre-mRNA splicing [11, 48–52]. Among these mechanisms, acting as miRNA sponges is currently the most extensively studied aspect for exon-derived circRNAs. Given that circCCDC85A is transcribed from the exon 2 of the host gene CCDC85A and localizes in the cytoplasm, we first hypothesized that circCCDC85A could function as an miRNA sponge. Next, we invalidated the hypothesis using bioinformatics analyses and a series of biological experiments, including CircNet online database, DIANA TOOLS, RIP, and luciferase reporter assay. Intriguingly, our experimental data agreed with the above hypothesis that circCCDC85A could indeed act as a molecular sponge of miR-550a-5p. Over-expression of circCCDC85A rescued miR-550a-5p-mediated promotion of the proliferative, migrative, and invasive abilities of breast cancer cells, and reversed miR-550a-5p-mediated downregulation of its target gene MOB1A. As described above, MOB1A reportedly inhibits tumor progression as a tumor suppressor in several cancer types. Consistently, we herein found that the enforced expression of MOB1A suppressed the proliferative, migrative, and invasive abilities of breast cancer cells. Our data presented the inhibitory function of circCCDC85A in breast cancer and suggested that circCCDC85A may regulate tumor progression at least in part by competitively activating MOB1A by serving as a sponge platform for miR-550a-5p.

Similar to our results, miR-550a-5p has been reported to function as a tumor promoter in colorectal cancer and lung adenocarcinoma [53, 54]. However, currently no literature report elucidates the biological function of miR-550a-5p in breast cancer. To the best of our understanding, this work is the first to reveal the effects of miR-550a-5p in breast cancer and supplements new information on the tumor-related function of miR-550a-5p. We found that miR-550a-5p could reinforce the proliferative, migrative, and invasive abilities of breast cancer cells through directly targeting 3’UTR of the tumor suppressor MOB1A. Intriguingly, our findings showed that the enforced expression of circCCDC85 could efficiently bind to miR-550a-5p as a ceRNA, and reversed miR-550a-5p-mediated enhanced cell proliferation, migration, and invasion.

However, in breast cancer, it is reported that circRNAs do not only work through miRNA sponges. For instance, Li et al. found that circ-HER2 encoded a novel protein HER2-103, which was crucial for triple-negative breast cancer (TNBC) cell proliferation and invasion, and its discovery identified that circ-HER2/HER2-103-expressing TNBC patients could benefit from the clinically approved HER2 antibody pertuzumab [23]. Xu et al. found that circSMARCA5 could bind to its parent gene locus, forming an R-loop, which results in transcriptional pausing at exon 15 of SMARCA5, thus inhibiting DNA damage repair to improve sensitivity to cytotoxic drugs in breast cancer [55]. Another circular RNA circSKA3 was identified to promote breast cancer progression by complexing with RNA-binding protein Tks5 and integrin β1, inducing invadopodium formation [56]. Chen et al. reported that FLI1 exon-derived circRNA could act as an upstream regulator to promote breast cancer metastasis by coordinately regulating DNA methylating enzyme DNMT1 and demethylating enzyme TET1 [39].

Although this study suggests that tumor suppressor circCCDC85A may regulate breast cancer progression by functioning as a miR-550a-5p sponge, it remains unclear whether circCCDC85A regulates tumor progression through other mechanisms, such as encoding proteins or interacting with RBPs. Accordingly, the role of this circRNA in breast cancer still needs further investigation, and we aim to proceed towards this issue in future studies.

**Fig. 7:**
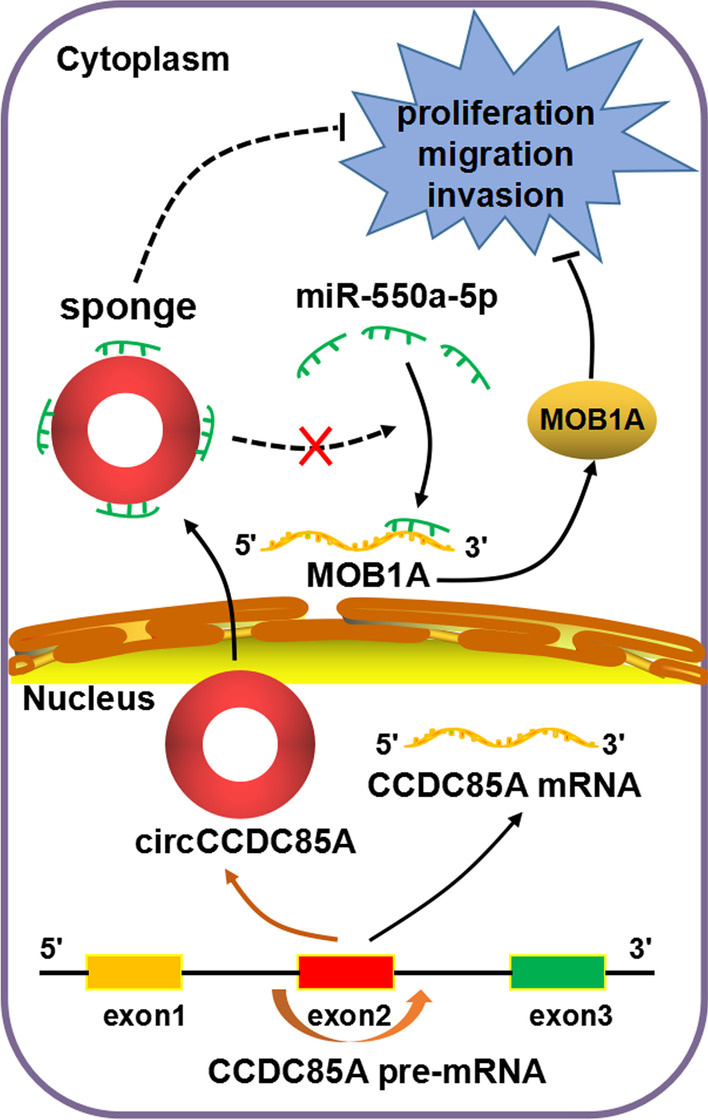
The mechanism diagram that circCCDC85A may inhibit breast cancer proliferation, migration and invasion via acting as a miR-550a-5p sponge to enhance MOB1A expression

This work demonstrated that circCCDC85A was downregulated in breast cancer and inhibits the proliferative, migrative, and invasive abilities of breast cancer cells. Mechanically, circCCDC85A may serve as a molecular sponge of miR-550a-5p and restore miR-550a-5p-mediated targeting repression of tumor suppressor gene MOB1A in breast cancer (Fig. 7). These findings support novel molecular evidence stating that circCCDC85A exerts biological function through the miR-550a-5p/MOB1A axis and provides a promising strategy for breast cancer treatment.

## Conclusions

This study showed that circular RNA circCCDC85A is downregulated in breast cancer and could inhibit the proliferation, migration, and invasion of breast cancer cells. Mechanically, circCCDC85A may serve as a molecular sponge of miR-550a-5p and restore miR-550a-5p-mediated targeting repression of the tumor suppressor gene MOB1A in breast cancer. These findings provide novel evidence that circCCDC85A inhibits the progression of breast cancer by functioning as a molecular sponge of miR-550a-5p to enhance MOB1A expression.

## Supplementary Information

**Additional file 1: Figure S1**. The predicted KEGG pathway ID, name, p-value, enriched gene count and gene name of miR-550a-5p through DIANA TOOLS.

**Additional file 2: Figure S2**. MOB1A inhibits cell proliferation, migration and invasion of breast cancer. A, The transfection efficacy of MOB1A overexpression plasmid in MDA-MB-231 cells was detected by qRT-PCR. B and C, Cell proliferation ability of MDA-MB-231 cells transfected with MOB1A overexpression plasmid or empty vector were evaluated by CCK-8 assay and colony formation assay. D-F, Cell migration and invasion abilities of MDA-MB-231 cells transfected with MOB1A overexpression plasmid or empty vector were evaluated by wound healing assay, transwell migration and invasion assay. *P<0.05. ***P<0.001.

**Additional file 3: Table S1**. The primers used in the PCR amplification..

## Data Availability

All data in our study are available upon request.

## Abbreviations

CircRNAs: Circular RNAs
miRNAs: MicroRNAs
ncRNAs: Non-coding RNAs
RNA-Seq: High-throughput RNA sequencing
FISH: Fluorescence in situ hybridization
qRT-PCR: Quantitative reverse transcription PCR
RIP: RNA-binding protein immunoprecipitation
KEGG: Kyoto Encyclopedia of Genes and Genomes
ceRNA: Competitive endogenous RNA
MRE: MiRNA response elements
