# Supplementary figures and images for: Circular RNA circCCDC85A inhibits breast cancer progression via acting as a miR-550a-5p sponge to enhance MOB1A expression
Source: Breast Cancer Res. 2022 Jan 4;24:1. doi: 10.1186/s13058-021-01497-6 (PMC8725284; doi:10.1186/s13058-021-01497-6)

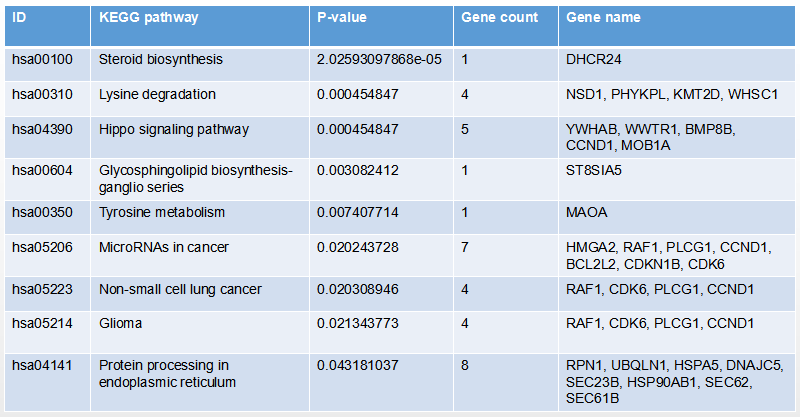

Supplement: Supplementary file 1 — Additional file 1: Figure S1. The predicted KEGG pathway ID, name, p-value, enriched gene count and gene name of miR-550a-5p through DIANA TOOLS. [file 13058_2021_1497_MOESM1_ESM.png]

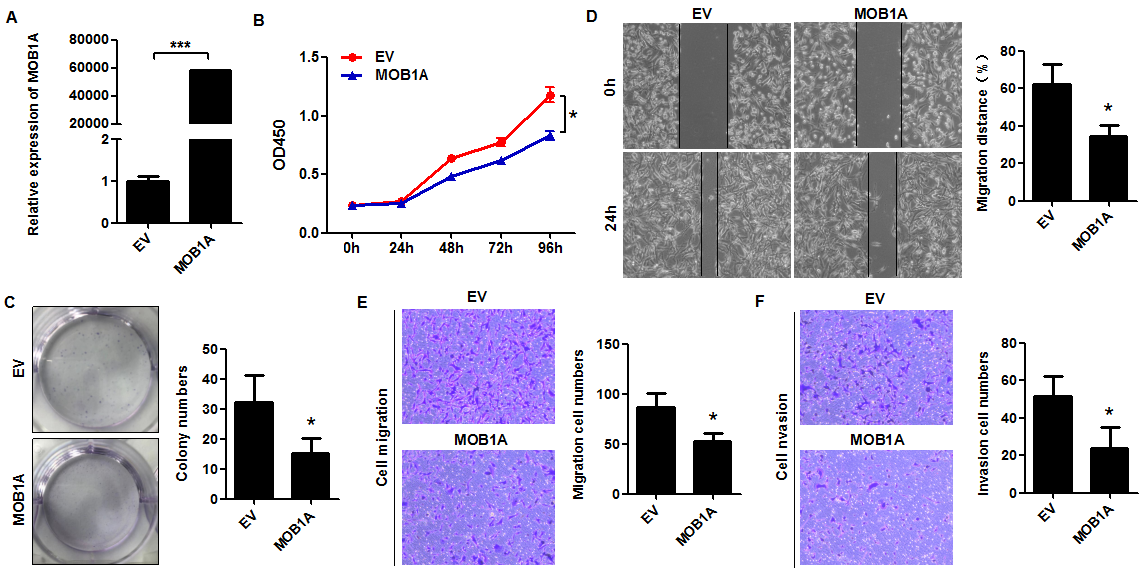

Supplement: Supplementary file 2 — Additional file 2: Figure S2. MOB1A inhibits cell proliferation, migration and invasion of breast cancer. A, The transfection efficacy of MOB1A overexpression plasmid in MDA-MB-231 cells was detected by qRT-PCR. B and C, Cell proliferation ability of MDA-MB-231 cells transfected with MOB1A overexpression plasmid or empty vector were evaluated by CCK-8 assay and colony formation assay. D-F, Cell migration and invasion abilities of MDA-MB-231 cells transfected with MOB1A overexpression plasmid or empty vector were evaluated by wound healing assay, transwell migration and invasion assay. *P<0.05. ***P<0.001. [file 13058_2021_1497_MOESM2_ESM.png]
