# Supplementary material for: Circular RNA circCCDC85A inhibits breast cancer progression via acting as a miR-550a-5p sponge to enhance MOB1A expression
Source: Breast Cancer Res. 2022 Jan 4;24:1. doi: 10.1186/s13058-021-01497-6 (PMC8725284; doi:10.1186/s13058-021-01497-6)
Supplement: Supplementary file 3 — Additional file 3: Table S1. The primers used in the PCR amplification. [file 13058_2021_1497_MOESM3_ESM.docx]

**Supplementary Table S1 The primers used in the PCR amplification**

| **Genes** | **Primers** |
| --- | --- |
| **CCDC85A (divergent)** | Forward, 5’-CCCCATCACCGGAATGTCTA-3’  Reverse, 5’-ACTTCCTCCTGCTTCACCTC-3’ |
| **CCDC85A (convergent)** | Forward, 5’-GACCTCTGCTGTTTCCTGGA-3’  Reverse, 5’-ACTTCCTCCTGCTTCACCTC-3’ |
| **MOB1A** | Forward, 5’-TCCCAGGTTCATGCCATTCT -3’  Reverse, 5’- ATCCTGGCTAACACGGTGAA-3’ |
| **GAPDH** | Forward, 5’-CGCTGAGTACGTCGTGGAGTC-3’  Reverse, 5’-GCTGATGATCTTGAGGCTGTTGTC-3’ |
| **U6** | RT, 5’-AACGCTTCACGAATTTGCGT-3’  Foward, 5’-CTCGCTTCGGCAGCACA-3’  Reverse, 5’-AACGCTTCACGAATTTGCGT-3’ |
